# Hypomania Risk in Noninvasive Brain Stimulation

**DOI:** 10.7759/cureus.2204

**Published:** 2018-02-19

**Authors:** Abhishek Gupta, Mahwish Adnan

**Affiliations:** 1 Geriatrics, Center for Addiction and Mental Health, University of Toronto; 2 Center for Addiction and Mental Health, University of Toronto

**Keywords:** rtms, tdcs, hypomania, mania, antidepressant, repetitive transcranial magnetic stimulation, transcranial direct current stimulation

## Abstract

Noninvasive brain stimulation, using electromagnetic waves (repetitive transcranial magnetic stimulation (rTMS)) and direct electrical current (transcranial direct current stimulation (tDCS)), is a new frontier in treating psychiatric maladies. While still being developed as viable treatment options, both options have had numerously reported side-effects, with hypomania being a significant concern during investigations. While there has been a relatively rare incidence of hypomania among rTMS/tDCS trials, it still posits an important issue regarding the safety of both treatment modalities. This review studies the reported episodes of hypomania in rTMS and tDCS trials in order to identify any patterns that may cause the same. Such patterns included higher stimulation strengths with long stimulation periods. These factors, if modified, along with an established regimen of screening and prophylaxis against hypomanic risks, may be effective protection against hypomania, as well as to prevent manic episodes.

## Introduction and background

Noninvasive brain stimulation is a novel frontier in the treatment of psychiatric pathology, especially depression. It is an umbrella term consisting of multiple methodologies, rTMS (repetitive transcranial magnetic stimulation) and tDCS (transcranial direct current stimulation) being the major forms of this new treatment [1-2]. While rTMS utilizes focal magnetic waves to stimulate specific neural foci, tDCS utilizes scalp electrodes to induce weak direct electrical stimulation of neural foci [3]. While traditional brain stimulation techniques, primarily electroconvulsive therapy (ECT), have often been effective in treating unipolar/bipolar depression, as well as acute mania, they have many restrictions [2-3]. ECT requires prior general anesthesia and seizure prophylaxis, in addition to side effects, such as amnesia, prolactin release, and general hypothalamic-pituitary axis (HPA) stimulation [2, 4-5]. rTMS/tDCS, although having side effects of their own, do not have such significant preparatory requirements for use.

Reported side effects of rTMS include seizures, hearing loss, headache, and scalp pain. Screening and prophylaxis for seizure risks have been a major recommendation for rTMS recipients. In addition, post-rTMS monitoring for 'kindling' or lowered seizure threshold is another routine recommendation commonly adopted in rTMS trials. While it is hypothesized and observed that lower frequency rTMS has a comparatively low incidence of seizure episodes, prophylaxis and monitoring for it remains a significant concern in rTMS recipients. This prophylaxis routinely includes establishing a stable therapeutic medication regimen that can be maintained prior to and throughout the rTMS course. In addition, motor cortical thresholds are established based on this regimen prior to and adjusted after rTMS stimulus. Similar precautions include earplugs against temporary increases in auditory thresholds and analgesics, along with spaced rTMS sessions, for any headache side-effect [2]. Therefore, rTMS has its own set of reported multiple side-effects that must be routinely screened and monitored for in its administration.

On the other hand, tDCS has its own side-effect profile. While tDCS has been shown to significantly stimulate neuron clusters in a focused manner, its effects are still dissipated to some extent by the skull. As such, it is preferable to ECT in terms of producing a focused neurostimulatory effect but not as much as rTMS. Further, tDCS has a significant advantage in lacking any seizure side-effects or 'kindling' effect, a severe limitation in rTMS applications. tDCS recipients have primarily reported skin manifestations at scalp electrode sites, most managed via topical skin preparations [3].  

However, while rTMS/tDCS trials in treating depression states in unipolar/bipolar depression have had promising results, hypomania is a reported side-effect in multiple instances [3]. For the purpose of this review, hypomania is defined and screened for according to its DSM-IV (Diagnostic and Statistical Manual - Fourth Edition) criteria in Table 1.

**Table 1:** DSM-IV Criteria for a Hypomanic Episode Table adapted from [6], Pg 485. DSM-IV: Diagnostic and Statistical Manual, Fourth Edition, Revised

The following is the DSM-IV criteria required for the diagnosis of hypomania:
1) A unique period of persistently elevated, expansive, or irritable mood, lasting throughout a minimum of four days, which is identifiably different from the usual non-depressed mood state
2) During the episode of mood disturbance, three or more of the following symptoms (four, if the mood is only irritable) have persisted and significantly present:
- Inflated self-esteem/grandiosity
- Decreased need for sleep
- More talkative than usual or pressure to keep talking
- Flight of Ideas or subjective experience that thoughts are racing
- Distractability
- Increase in goal-directed activity or psychomotor agitation
- Excessive involvement in pleasurable activities that have a high potential for painful consequences
3) The hypomanic episode is associated with a significant change in functioning that is uncharacteristic of the person when not symptomatic
4) The disturbance in mood and the change in functioning are observable by others
5) The hypomanic episode is not severe enough to cause marked impairment in social or occupational functioning or to necessitate hospitalization, and there are no psychotic features
6) The symptoms are not due to the direct physiological effects of a substance (e.g., a drug of abuse, a medication, or other treatment) or a general medical condition (e.g., hyperthyroidism)

## Review

Summary of adverse events

Hypomanic events reported in rTMS often developed in a variety of patients. A summary of reported hypomanic events has been provided in Table 2.

**Table 2:** Hypomanic Events Reported in rTMS Administration * Refers to number of sessions received before developing hypomania ** Refers to number of antidepressants being co-administered at the time of rTMS sessions LDLPFC: left dorsolateral prefrontal cortex; Hz: Hertz (frequency of rTMS administered); PTSD: post-traumatic stress disorder

Study/Trial	Patients	Settings	Number of sessions*/Site	Number of antidepressants**
Nedjat et al.	3 patients (healthy)	2 patients – 10 Hz	1 / Unknown	0
		1 patient – 20 Hz	1 / Unknown	0
Krstic et al.	1 patient (bipolar disorder)	Unknown	Unknown / LDLPFC	1
Ozten et al.	4 patients (unipolar depression)	1 patient – 25 Hz	9 / LDLPFC	1
		1 patient – 25 Hz	14 / LDLPFC	1
		1 patient – 25 Hz	12 / LDLPFC	1
		1 patient – 25 Hz	12 / LDLPFC	2
Philip et al.	1 patient (unipolar depression and PTSD)	1 patient – 5 Hz	6 / LDLPFC	0

Nedjat et al. reported three individuals out of a total sample of 50 individuals receiving rTMS, to have developed hypomanic symptoms after the very first session [7]. These individuals' hypomanic symptoms began 60 minutes after a rTMS treatment session, resolving the next day. Meanwhile, other reported hypomanic cases often received multiple rTMS sessions at high stimulation frequencies ranging from 20-25 Hz.

Krstić et al. reported a unique case of an individual with treatment-resistant depression who developed hypomanic symptoms on rTMS administration at an unknown strength [8]. However, it is important to note that this individual was also receiving concurrent partial sleep deprivation therapy in addition to her antidepressant regimen. Her management of hypomania, while similar to the above cases, did include some significant unique deviations in her scenario from other cases of hypomania in rTMS recipients discussed above. Rather than receive contralateral cerebral rTMS in the rostrolateral prefrontal cortex (RLPFC), rTMS was discontinued altogether to achieve euthymia. The subsequent diagnosis of bipolar disorder was also a significant factor, unique from the unipolar depression in other reported cases [8].

Ozten et al. reported four cases of hypomanic symptoms in individuals with treatment-resistant depression, all of whom had experienced one unsuccessful trial of an antidepressant course [9]. All four cases experienced hypomania after 25 Hz rTMS exposure to the L-DLPFC (left dorsolateral prefrontal cortex). Subsequently, medication regimens were adjusted and a switch was made in all four cases from L-DLPFC to R-DLPFC (right dorsolateral prefrontal cortex) rTMS stimulation at 1 Hz [9]. As such, Ozten et al. reported achieving a euthymic status after six to 11 sessions in these individuals or R-DLPFC stimulation.

Philip et al. mentioned a similar case where hypomanic episodes were observed while receiving rTMS stimulation at a strength less than 10 Hz [10]. This is significant since lower frequency stimulations have been generally deemed safer in rTMS recipients, even regarding psychiatric complications [2]. In addition, her similarly complicated psychiatric history due to PTSD (post-traumatic stress disorder), in addition to a history of treatment-resistant depression, is a significant factor. Further, this patient had previously received electroconvulsive therapy regimens, along with numerous trials of antidepressant regimens. 

Subsequently, a summary of the observed hypomanic cases in tDCS trials has been provided in Table 3.

**Table 3:** Hypomanic Events Reported in tDCS Administration Table adapted from [1], Pg 15 mA: milliampere; tDCS:  transcranial direct current stimulation

Study/Trial	Patient	Settings	Session duration (minutes)	Number of sessions	Adverse Event
Arul-Anandam et al. [11]	1 patient (unipolar depression)	2 mA, 0.06 mA/cm^2^	30	10	Hypomania
Baccaro et al. [12]	1 patient (unipolar depression)	2 mA, 0.06 mA/cm^2^	30	5	Hypomania
Galvez et al. [13]	1 patient (bipolar depression)	2 mA	20	14	Hypomania
Brunnoni et al. 2013 [14]	6 patients (unipolar depression)	2 mA, 0.08 mA/cm^2^	30	12	4 Hypomania/ 2 Mania
Pereira Junior Bde et al. [15]	1 patient (bipolar depression)	2 mA, 0.08 mA/cm^2^	30	12	Hypomania

Arul-Anandam et al. reported one of the first incidences of hypomania in a tDCS recipient in 2010 [11]. This study reported hypomania in a 57-year-old male with a history of major depression disorder (MDD), who received alternating courses of placebo (sham) and active tDCS at the LDLPFC area. The individual reported racing thought patterns after the first eight tDCS session which lasted less than four days. However, upon the tenth session, sufficiently prolonged and severe symptoms developed to be classified as hypomanic behavior. This case study, as part of a larger trial, also made a critical observation that sham (placebo) tDCS often produced very transient hypomania-like symptoms whereas active tDCS resulted in an actual hypomanic episode [11].

Similarly, Baccaro et al., 2010 [12] reported a singular case of hypomanic behavior in a 58-year-old male with a history of MDD and very transient spontaneous mood elevation episodes. Though this individual's tDCS sessions were temporarily suspended and later resumed at lower stimulus strength, it is noted that complete tDCS discontinuation was required to achieve euthymia. Additionally, this case study stressed the importance of using specific mental assessment scales designed for hypomania to estimate actual mood state instead of a patient's own subjectively reported mood state (such as in Arul-Anandam et al., 2010 [11]) [12]. These scales include Hamilton Depression Rating Scale (HDRS), Montgomery-Asberg Depression Rating Scale (MADRS) and Young Mania Rating Scale (YMRS), all three of which were used to estimate the hypomanic state in this case study. However, like the above case study [11], Baccaro, et al., [12] also arrived at a similar conclusion that tDCS posits a possible hypomanic risk in bipolar disorder patients specifically.

The hypomanic incident reported by Galvaez et al., [13] in a 33-year-old female with a history of bipolar disorder, highlighted a significant observation, unique from other similar incidents. The trial noticed that her tDCS electrodes were shifted from bifrontal positions to frontoextracephalic positions, which was the only difference in the tDCS protocol before and after developing hypomanic symptoms. Although the two tDCS sessions with different configurations were separated by a period of more than seven months, Galvaez et al., suspected that the two configurations had hypomanic properties. The prior bifrontal tDCS was hypothesized to have a 'priming' effect favoring hypomania while frontoextracephalic tDCS stimulation of deeper brain structures further increased the same hypomanic risk. Though only at hypothetical stage, the trial did concur with the above cases mentioned here that bipolar disorder afflicted tDCS recipients should be closely monitored and prophylactically managed for hypomanic behavior, especially when frontoextracephalic area stimulation is involved [13].

Brunnoni et al., [14], reported on a tDCS trial consisting of 82 participants with unipolar or bipolar depression. Though significantly improved clinical outcomes were observed, based on HDRS and BDI (Beck Depression Inventory), four reported cases of hypomanic behavior were observed. The trial also noticed that antidepressants, especially with serotonergic action, often increased tDCS stimulatory effects [14]. This observation may underline the pathophysiology behind the hypomanic induction effect observed in the tDCS cases discussed above.

Discussion

The hypomanic events observed in rTMS-tDCS scenarios were observed to have many distinct patterns. tDCS induced hypomania occurred in those receiving at least two mA ( mili -ampere), a relatively high stimulation threshold. In addition, a range of 20-30 minute sessions was observed in the same cases [15]. Comparatively, for rTMS, while the majority of these individuals received high-frequency stimulation at L-DLPFC, it is not a universal finding in all reported cases [16]. Secondly, multiple sessions (five or more) along with a relatively high frequency (25Hz) were also common in a majority of the cases observed. Similar to tDCS administered cases, most hypomanic episodes in rTMS recipients were observed in individuals with a history of unipolar or bipolar depression.

While the above patterns are evidence of some predisposing conditions to hypomania, it is important to note that hypomania itself is a rare occurrence in rTMS/tDCS trials. Prior studies have hypothesized for hypomania to be a significant risk in patients with bipolar disorder despite concurrent mood stabilizers being administered. As such, close monitoring is recommended for the emergence of hypomania, in addition to any prophylactic mood stabilizers in bipolar disorder-afflicted recipients of rTMS [2]. Similarly, ongoing trial parameters responsible for hypomania incidents often involve high stimulation strengths and long stimulation periods via multiple sessions.

Subsequently, this presents an initial reference point for future investigations and trial designs in hypomanic individuals receiving rTMS/tDCS. The majority of trials that experienced hypomanic events had specific inclusion criteria for bipolar or unipolar depression patients. This is a critical step and, therefore, a strong recommendation for future trials to screen for undiagnosed bipolar disorder in participants with depression. Possible avenues in future investigations include using a lower stimulation frequency and strength for rTMS/tDCS. Further, greater screening among trial participants for hypomanic risks, including bipolar mania and lack of concurrent mood stabilizer use, is another possibility. In addition, if trial parameters cannot be adjusted to lower stimulation, greater surveillance for hypomanic/manic symptoms can be instituted. For example, many trials universally estimated a participant's progress with psychological assessments sensitive for depression, such as the  Montgomery-Åsberg Depression Rating Scale (MADRS), the Hamilton Rating Scale for Depression (HRSD), and the Beck Depression Inventory (BDI). However, the use of mania-sensitive assessments, such as the Young Mania Rating Scale (YMRS), was not a part of the trial structure in many cases. As such, it can also be recommended that the YMRS or the Hypomania/Mania Symptom Checklist (HCL-32) be adapted into rTMS-tDCS trials in order to screen for hypomanic symptoms alongside depression severity assessments.

For management, complete cessation of rTMS has been an effective strategy for hypomanic symptoms so far. However, such a strategy has not been discussed in any of the literature reviewed for tDCS. Lastly, concurrent antidepressant use has been also been implicated and, as such, is a modifiable factor for hypomania in both rTMS and tDCS. Therefore, hypomanic risks may be attenuated via greater preliminary and post-procedure risk screening, as well as implementing lower stimulation frequency and strength in trials.

From an endocrine perspective, post-session surveillance via a serum thyroid function profile may also be warranted [5, 15]. While endocrine disturbances have not been linked to the development of hypomania, it may be a contributing factor nonetheless. Future investigations would also be recommended to distinctively diagnose hypomanic and manic incidents in rTMS/tDCS trials as they are unique and distinct pathologies. Preliminary surveillance of hypomania and the unique clinical conditions prior to its development may contribute to developing significant precautionary protocols for curtailing the risk of completely developing manic symptoms in rTMS/tDCS recipients.

The above review and subsequent recommendations have considerable limitations. Since rTMS and tDCS themselves are technologies in developmental stages, limited knowledge regarding ideal stimulation strength, session duration, and frequency is a significant unknown factor. As such, a safe threshold to prevent side effects, such as hypomania, is also yet to be discovered. Reported cases have led to the sporadic implementation of screening protocols for hypomania/mania in bipolar disorder-affected individuals for rTMS [2]. However, there is a lack of similar protocols for unipolar depression or various other neuropathologies currently being tested for rTMS/tDCS treatment. This may leave a significant gap in the literature on reported cases involving hypomania in noninvasive stimulation. To summarize, the relatively low incidence of hypomania-related literature for rTMS/tDCS limits the significance of the above recommendations.

## Conclusions

rTMS and tDCS are novel strategies for combating various psychiatric and neurological disorders. Still in development, their side effect and safety profile has yet to be completely explored. As such, hypomania is a serious consideration in anyone receiving rTMS or tDCS as a potential treatment. While it is currently a relatively uncommon phenomenon, establishing the predisposing risks and subsequent management should be a significant priority for future rTMS/tDCS trials. Based on the currently limited body of literature available, this review can suggest future trials to adopt greater screening and medication prophylaxis in bipolar disorder-afflicted recipients of rTMS/tDCS. Conclusively, however, this review cannot state any concrete ideal parameters for rTMS/tDCS administration to successfully avoid hypomania in its recipients. Yet, it is noted that there is a greater need for future investigations to adopt comprehensive screening protocols for hypomanic risks. Adoption of such protocols may prevent hypomanic/manic episodes, thereby improving rTMS/tDCS overall as viable therapies.
